# Fast Neutron Measurement System Using Prompt Gamma Neutron Activation Solid Converter: Monte Carlo Study

**DOI:** 10.3390/s23084133

**Published:** 2023-04-20

**Authors:** Jonathan Walg, Itzhak Orion

**Affiliations:** Department of Nuclear Engineering, Ben-Gurion University of the Negev, Beer-Sheva 84105, Israel; walg@post.bgu.ac.il

**Keywords:** PGNA, KCl, radiation, MCNP, accelerator, converter

## Abstract

Measuring fast neutron emission around accelerators is important for purposes of environmental monitoring and radiation safety. It is necessary to detect two types of neutrons: thermal and fast. Fast neutron spectroscopy is commonly employed using a hydrogen-recoil proportional-counter; however, its threshold is 2 MeV. The aim of this study was to expand PGNA converters based on KCl to fulfil the need to detect neutron energies ranging from 0.02 MeV to 3 MeV. In our previous research, we established a counting system comprised of a large converter of KCl with a NaI(Tl) gamma radiation spectrometer. The KCl converter is efficient for fast neutron prompt gamma emission. The potassium naturally includes a radioisotope that emits 1.460 MeV gamma rays. The presence of the constant level of 1.460 MeV gamma ray counts offers an advantage, providing a stable background for the detector. The study was carried out using MCNP simulations of the counting system with a variety of PGNA converters based on KCl. We concluded that KCl mixtures combined with other elements, such as PGNA converters, demonstrated improved detection performance for fast neutron emissions. Furthermore, an explication of how to add materials to KCl to provide a proper converter for fast neutrons was introduced.

## 1. Introduction

The increment of using devices for measuring fast neutrons is mainly due to the growing application of proton accelerators to produce medical radioisotopes for imaging as well as the use of energetic proton beams in radiotherapy. Therefore, it is increasingly vital to measure fast neutrons even when the flux is not high, or far from the accelerator.

Measuring fast neutron emission around accelerators is of great importance for purposes of environmental monitoring and radiation safety. Neutrons are divided into three vast energy groups: thermal, epithermal, and fast neutrons. To measure the thermal neutrons, the BF_3_ gas ion chamber proportional counter is the most common among the variety of detectors [1]. Fast neutrons could be measured by two major detection methods. The first is a direct measurement of online signals produced by fast neutrons, and the second involves moderating fast neutrons toward thermal neutrons. The usage of a moderator for fast neutron detection can be complicated in terms of analyzing measurements due to complex interactions. There are several detectors for fast neutrons based on gamma radiation measurements from gamma induced by neutron reactions in a converter [2,3,4,5]. The gamma radiation detector produces a signal (after a certain time) from the neutron reaction, and subsequently the neutron flux can be obtained by analyzing the gamma counts [6,7,8,9,10,11,12,13].

**Figure 1:**
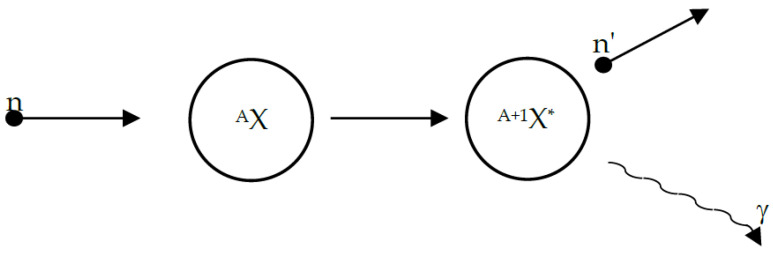
INS reaction illustration when a neutron is absorbed by a nucleus and a new neutron and gamma ray are emitted (* notes an excited nucleus).

Prompt gamma neutron activation (PGNA) analysis is a method whereby secondary gamma rays are measured. PGNA is a phenomenon that occurs when a neutron undergoes inelastic scattering (INS) in the nucleus. PGNA has been introduced into several applications for elemental identification. Notably, each isotope has its proper threshold energy, and only neutrons with this energy or above can perform INS. PGNA occurs when a fast neutron is absorbed by the nucleus, and the excited nucleus emits a neutron with a lower kinetic energy; as a result, the original nucleus has excess energy, which is instantly emitted as gamma rays and reaches its ground state [14,15,16]. The following illustration shows the reaction that occurs in a nucleus during an INS reaction (Figure 1).

Using the PGNA method for measuring fast neutrons offers an advantage. Indeed, the gamma radiation can be measured online, counted by a gamma radiation detector and rapidly analyzed since the INS reaction is prompt. The PGNA method enables researchers to identify a source of neutrons because the gamma radiation flux depends on the intensity of the neutron source; thus, using the gamma radiation spectrum makes it possible to determine the neutron’s energy range and its intensity [2,17]. As the neutron flux increases, a corresponding increase in gamma radiation emission occurs, leading to the detection of additional counts.

In this study, we perform Monte Carlo simulations of fast neutrons based on the PGNA method using the MCNP code system.

## 2. Materials and Methods

KCl was used as a PGNA converter with an NaI(Tl) gamma spectrometer and was deemed suitable for fast neutron detection [18]. The K-40 radioisotope presence (which occurs naturally in KCl) emits a constant level of gamma radiation that provides a stable background for the detector. Therefore, background changes due to environmental conditions are limited. This study adopted the KCl experimental setup for simulations and includes with the KCl salt a variety of additional materials to expand the neutron detection to encompass other energy ranges.

**Figure 2:**
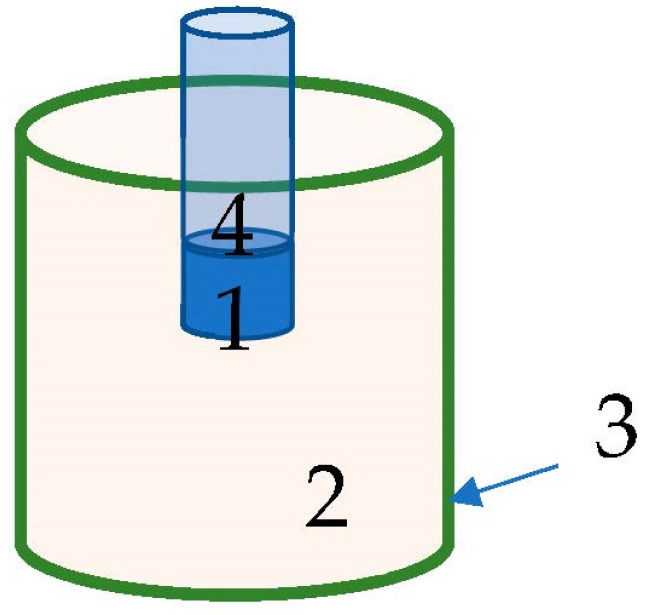
Description of the simulated measurement system that includes a converter where 1. is a 3″ × 3″ NaI gamma radiation detector; 2. is the PGNA converter; 3. is a cylindrical container for the converter with dimensions of 30 cm diameter and 36 cm height; 4. is the electronic part of the detector (taken as a vacuum in the simulation). A neutron source was placed at a distance of 25 cm from the detector’s center.

The MCNP code system version 5 [19] was utilized for the simulations. The geometry was input as illustrated in Figure 2. The materials used in the input were: NaI for the detector, Polymethyl methacrylate (PMMA) for the container, converter materials (changeable), and the room filled with air. The neutron source was defined as a unidirectional beam emitted 25 cm from the center of the container from a uniform rectangular surface of 36 cm × 30 cm. The aim of the neutron source was to represent reaching flux emitted from a proton accelerator, and a monoenergetic source was defined as between 0.2 and 3.0 MeV in order to inspect detectability vis-a-vis neutron energy. The converter materials used in the simulations were a mixture of 20% weight of the selected material from Table 1, and according to the natural abundance of Chlorine isotopes, the Cl-35 was 36.176% and Cl-37 was 11.424% of the atomic mass; the remaining 52.4% of the weight was comprised of natural potassium.

Cross sections for the converter elements that were found to have a good performance for PGNA were “.62C” series for the natural Potassium, “.61C” series for both Chlorine isotopes, and the cross sections series of the selected materials are presented in Table 1. The GPD label (photon-production data) in the list of cross sections indicates that the MCNP library includes the appropriate nuclear data.

Table 1 presents the cross sections and the evaluated nuclear data files used by MCNP. The suffix LLNL describes nuclear data libraries compiled by the Nuclear Data Group at Lawrence Livermore National Laboratory, and the suffix B-VI pertains to the National Nuclear Data Center at Brookhaven National Laboratory.

Output included the spectral detector response to gamma radiation emitted by PGNA and absorbed in the detector, using Tally type F8.

Energies up to 3 MeV were simulated due to the limited gamma detector volume, which did not exceed the size of 3″ × 3″.

## 3. Results

The gamma radiation measurement obtained by the NaI(Tl) detector after PGNA was performed using simulations with 10^8^ histories each. Every MCNP simulation included the NaI(Tl) detector cell response function that provides a gamma rays spectral result of pulse height distribution per incident neutron, hence the spectrum is normalized to number-of-histories.

**Figure 3:**
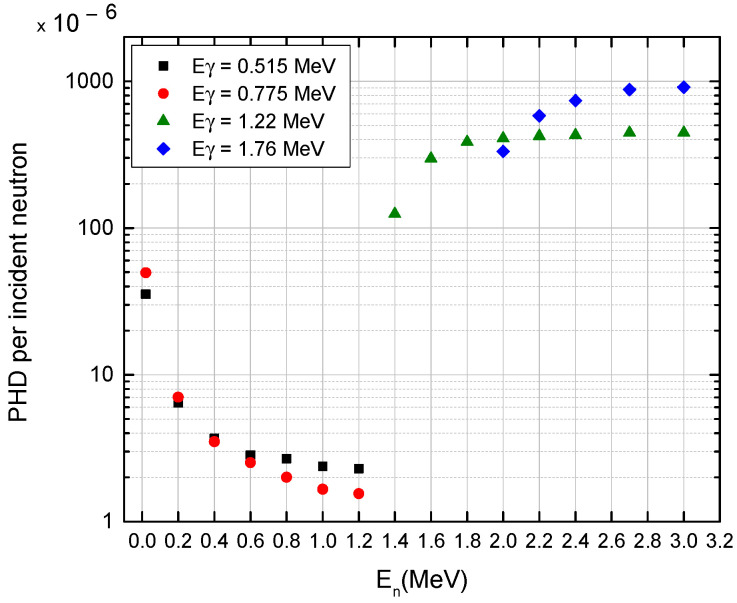
Summary of the intensity of the observed gamma radiation response (PHD per incident neutron) vs. neutron energy of KCl. Each color in the graph is the outcome of a different gamma energy line.

A summary of the PGNA simulation results after irradiating KCl salt versus different neutrons’ energy flux is presented in Figure 3. Four gamma-ray peaks were obtained and analyzed, demonstrating a change of regime between neutron energies 1.2–1.4 MeV and 1.2–2.0 MeV. From thermal neutron up to 1.2 MeV, the counts-per-incident-neutron of two gamma peaks at 0.515 MeV and at 0.775 MeV decreased by one order of magnitude. Gamma rays of E_γ_ = 1.22 MeV were detected above a neutron energy threshold of about 1.4 MeV raising to a plateau-line starting around 1.8 MeV. Gamma rays of E_γ_ = 1.76 MeV were detected above a neutron energy threshold of about 2 MeV and continuously rose.

**Figure 4:**
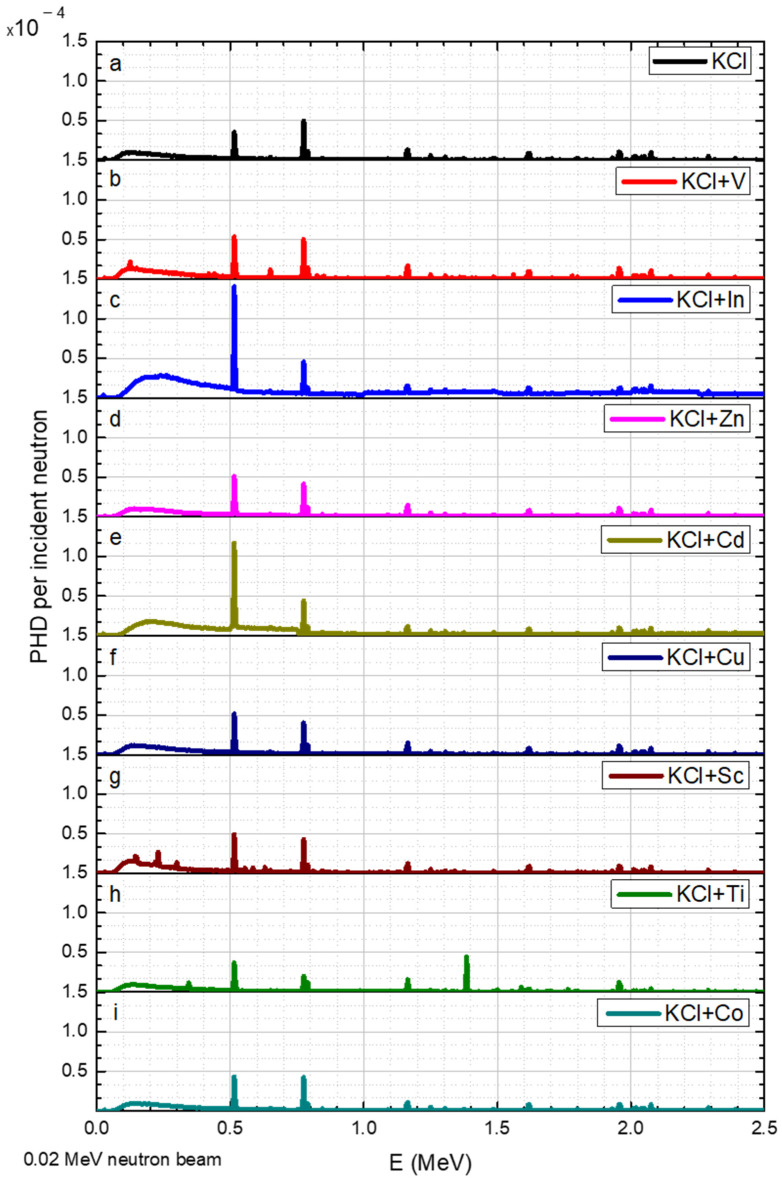
PGNA results for 0.02 MeV neutron energy, where the y axis is the gamma pulse height distribution (PHD) per incident neutron obtained in the detector and the x axis is the gamma radiation energy. The data corresponds to a range of elements (**a**–**i**) added to the KCl.

**Figure 5:**
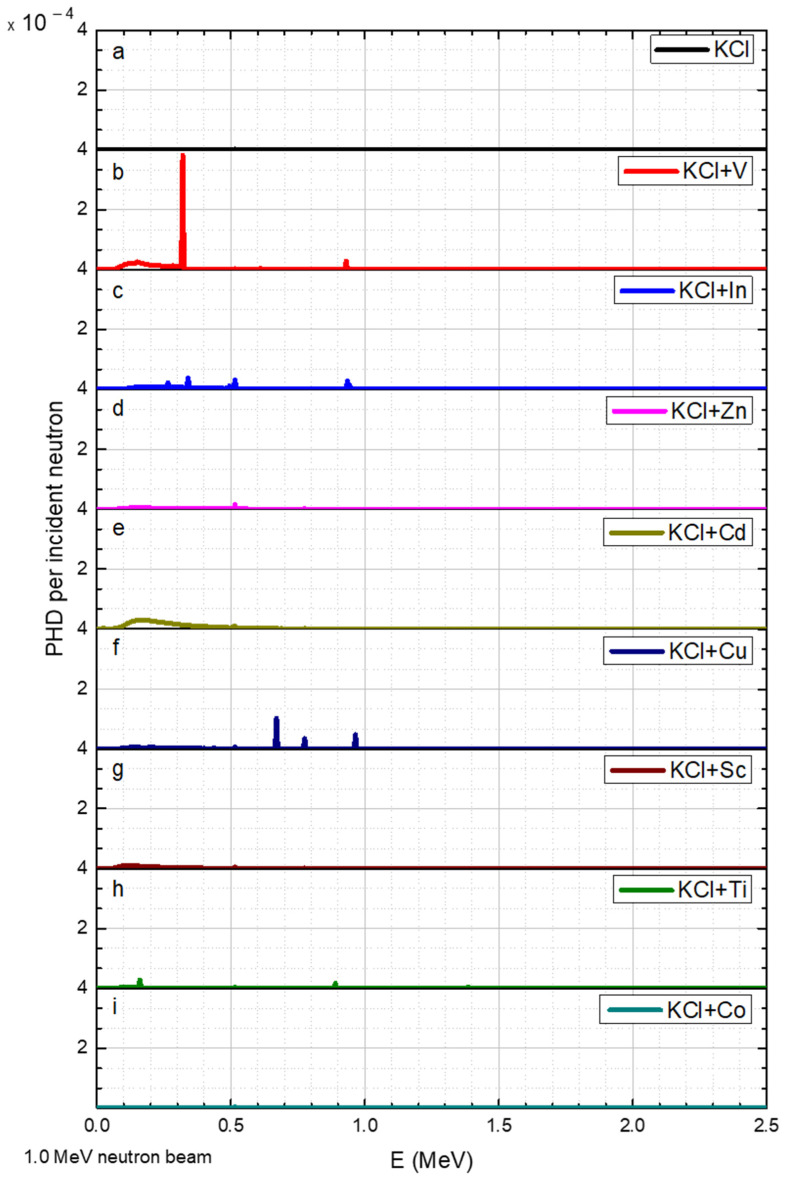
Neutron energy of 1.0 MeV PGNA results, where the y axis is the gamma pulse height distribution (PHD) per incident neutron obtained in the detector and the x axis is the gamma radiation energy. The data corresponds to a range of elements (**a**–**i**) added to the KCl.

**Figure 6:**
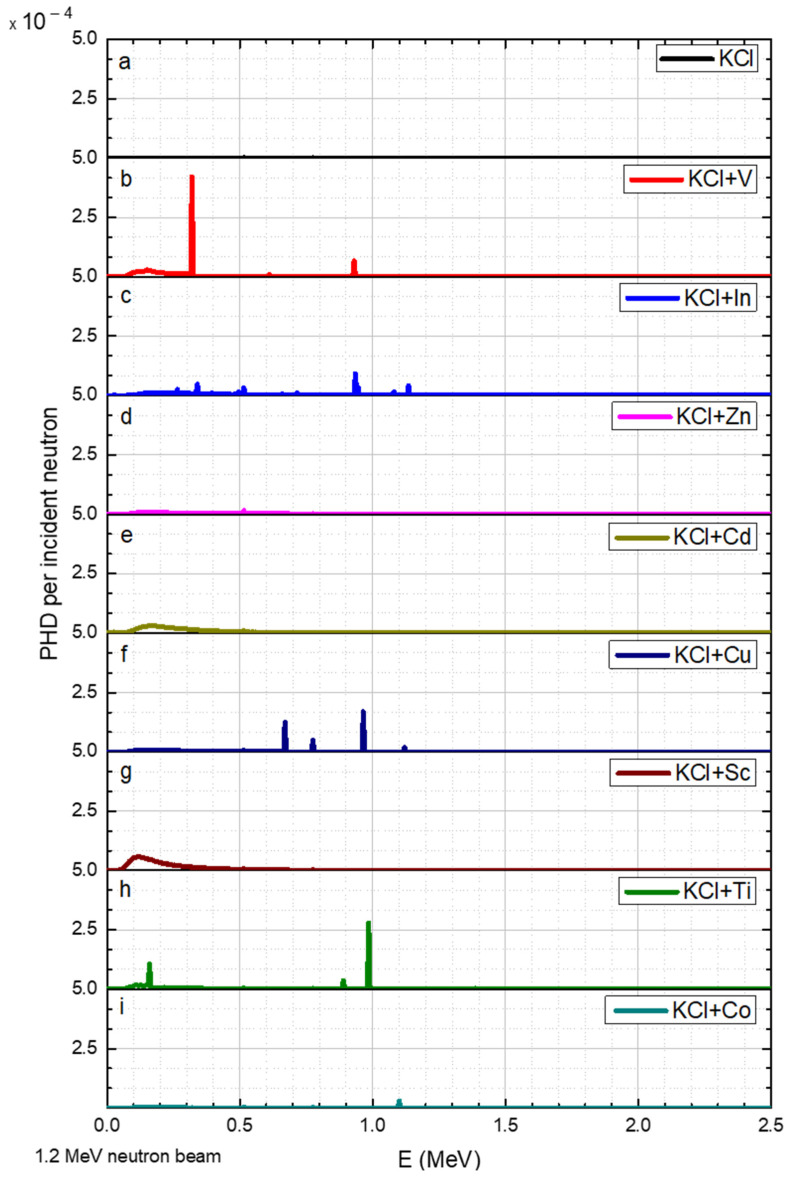
1.2 MeV neutron energy of the PGNA results, where the y axis is the gamma pulse height distribution (PHD) per incident neutron obtained in the detector and the x axis is the gamma radiation energy. The data corresponds to a range of elements (**a**–**i**) added to the KCl.

**Figure 7:**
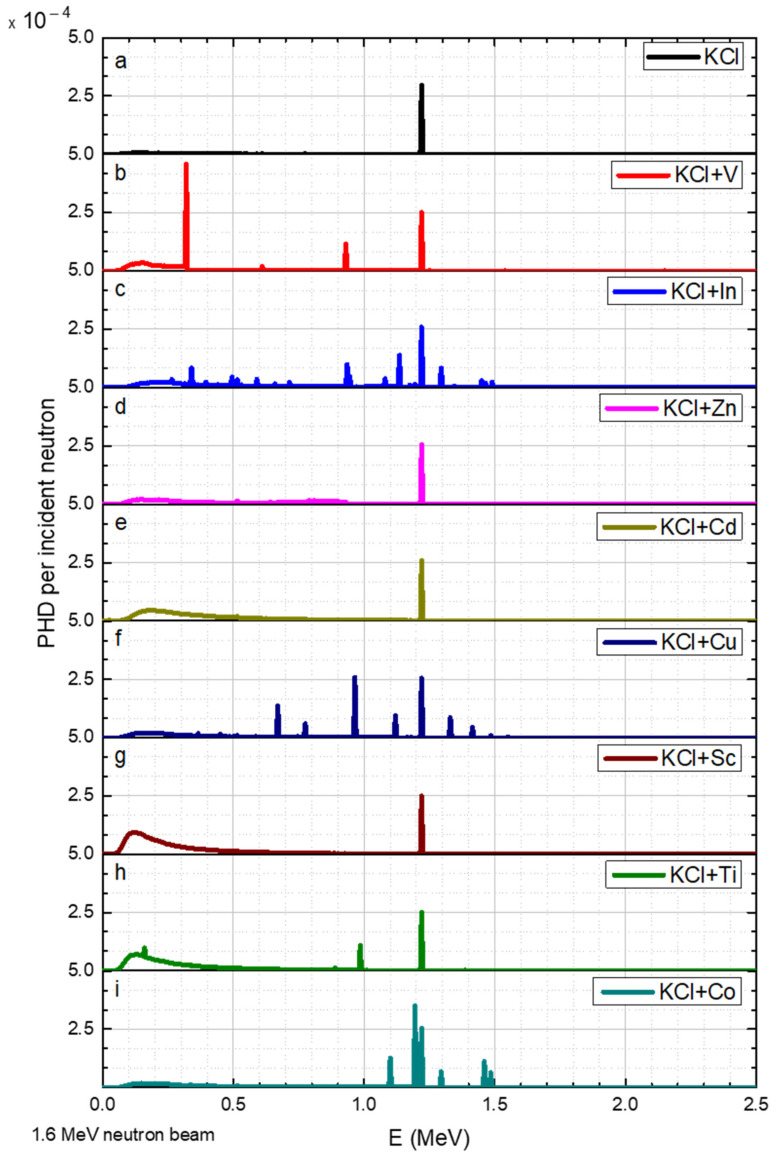
Irradiation of KCL salt and its elements with 1.6 MeV neutron, where the y axis is the gamma pulse height distribution (PHD) per incident neutron obtained in the detector and the x axis is the gamma radiation energy. The data corresponds to a range of elements (**a**–**i**) added to the KCl.

**Figure 8:**
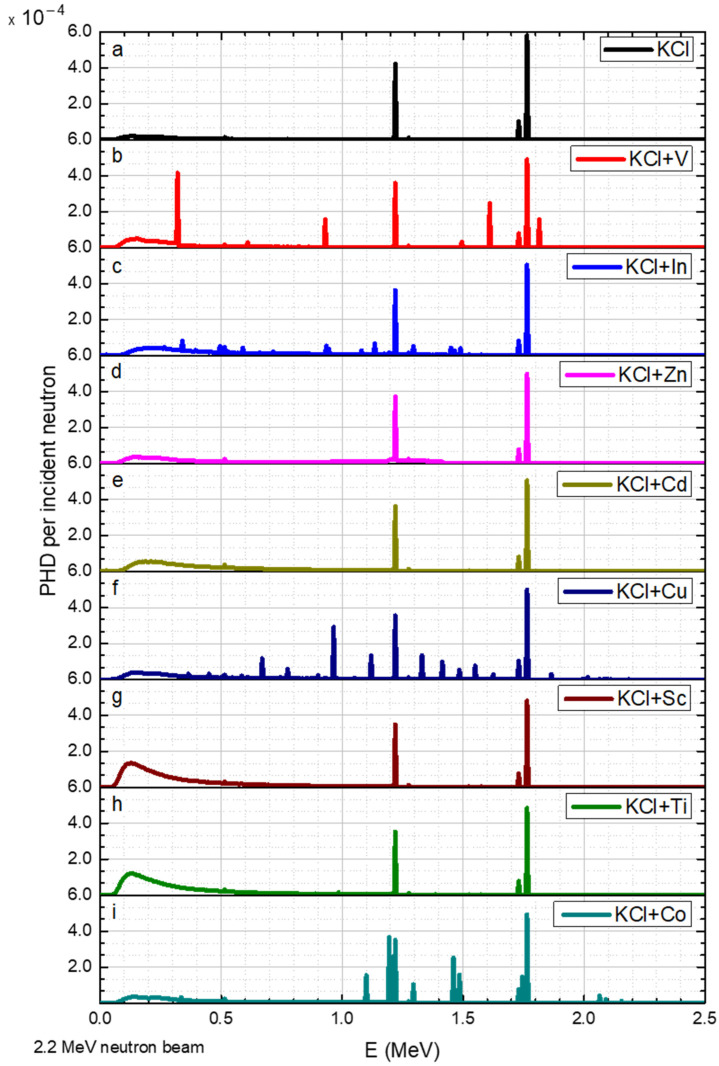
2.2 MeV neutron radiation on KCL salt and its elements, where the y axis is the gamma pulse height distribution (PHD) per incident neutron obtained in the detector and the x axis is the gamma radiation energy. The data corresponds to a range of elements (**a**–**i**) added to the KCl.

**Figure 9:**
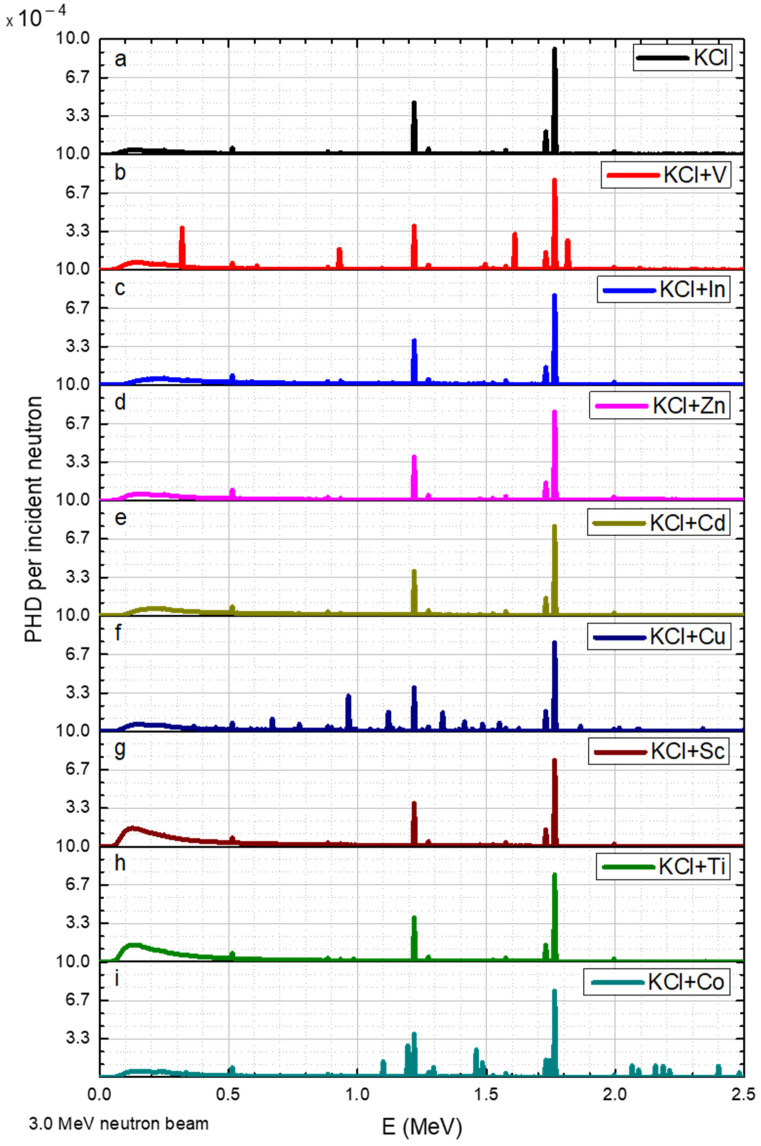
3.0 MeV radiation of neutrons on KCL salt and its elements, where the y axis is the gamma pulse height distribution (PHD) per incident neutron obtained in the detector and the x axis is the gamma radiation energy. The data corresponds to a range of elements (**a**–**i**) added to the KCl.

The results for neutron beams with discrete energies of 0.02, 1.0, 1.2, 1.6, 2.2, and 3.0 MeV are shown in Figure 4, Figure 5, Figure 6, Figure 7, Figure 8 and Figure 9, respectively.

The KCl converter displays gamma radiation counts at energies of 0.55 MeV and 0.775 MeV (Figure 4a). Adding 20% (wt) In or Cd to the KCl salt has almost no effect on the detection peak position of the KCl salt; nevertheless, the peak at 0.55 keV is three times higher for In and two times higher for Cd. When adding 20% of Ti or Sc to the KCl salt, the converter exhibits new peaks at 1.385 MeV and 0.23 MeV, respectively. The elements Co, Zn, Cu, and V showed similar peaks to the original KCl spectrum.

A 1.0 MeV neutron flux throughout the KCl converter had a minor influence, evident in Figure 3. The gamma pulse height distribution (PHD) per incident neutron results are shown with a specific range, according to the highest gamma peak in a given neutron energy. For example, adding V to the converter exhibited a gamma peak at 0.23 MeV (Figure 5b), and adding Cu obtained new peaks at 0.67, 0.775, and 0.965 MeV (Figure 5f). Furthermore, the presence of In in this neutron energy reduced the intensity of the peaks by a factor of two (Figure 5c), compared to the intensity in 0.02 MeV neutrons (Figure 4c). Additionally, Cd, Sc, and Ti were not efficient for 1 MeV neutrons.

Similarly, a 1.2 MeV neutron flux also slightly influences the KCl. Additionally, the peaks of V were slightly enhanced, while for In, a new peak was observed at 1.135 MeV (Figure 6b,c, respectively). As for Ti and Cu, the intensity of the peaks at 0.16 MeV and 0.985 MeV increased (Figure 6h,f, respectively) compared to their intensity at 1.0 MeV neutron. Adding Cd, Sc, Zn, or Co elements to the KCl did not improve the detection of 1.2 MeV neutrons.

As mentioned before, all the simulations contained a portion of KCl (80%). This explains the peaks at 1.22 MeV that appear in all graphs in Figure 7: the INS threshold for Cl-35 is 1.254 MeV [20]. Moreover, 1.6 MeV neutrons obtained the following differences (compared to a 1.2 MeV neutron beam): for In, a series of peaks appeared between 0.935 MeV and 1.49 MeV (Figure 7c). The gamma peak at 0.985 MeV observed in Ti 1.2 MeV (Figure 6h) was about three times smaller than the peak at 1.6 MeV (Figure 7h), while the peak observed at an energy of 0.16 MeV was broader (Figure 7h). For Co, new peaks were observed at 1.1 MeV, 1.195 MeV, and 1.46 MeV (Figure 7i). Cu also exhibited new peaks between 1.12 MeV and 1.415 MeV (Figure 7f).

A 2.2 MeV neutron obtained the following differences (compared to the results for 1.6 MeV): the Cl-35 peak at 1.22 MeV slightly increased and a new peak was observed at 1.765 MeV (Figure 8a). V exhibited new peaks at 0.93 MeV and at 1.61 MeV (Figure 8b). The peaks that appeared for In decreased slightly (Figure 7c).

Neutrons with an energy of 3 MeV resulted in several additional peaks for Co between 2 and 2.5 MeV.

The addition of Sc to the converter obtained a broad peak at ca. 0.25 MeV. This broad peak may enable the detection of neutrons, albeit without the ability to determine the neutron energy.

## 4. Discussion

The simulated system used in this experiment was based on the results of previous measurements of fast neutrons using KCl [18]. The improvement of the detection capability of the KCl system was tested by adding to the KCl various metals with a relatively high probability of undergoing INS. Table 2 summarizes the metals with the prospect of increasing/non-impairing the detection efficacy of the KCl converter (marked as Yes or No, respectively).

The application of these results in neutron measurement systems will enable researchers to determine the energy range of the neutron beam that reaches the converter.

The spectral results obtained in this study (shown in Figure 4, Figure 5, Figure 6, Figure 7, Figure 8 and Figure 9) indicate the presence of multiple gamma lines with varying energies. The moderate spectral resolution of the NaI(Tl) 3″ × 3″ detector could broaden these peaks in practical application. According to the literature, the resolution at 122 keV was around 10–11%; at 622 keV, it was around 6–7%; and at 2750 keV, it was around 3.7% [21]. Consequently, the measured peaks may overlap with one another. Nonetheless, for the intended purpose of this application, groups of peaks that cannot be distinguished from each other can still satisfy the method’s requirements. In these particular instances, peak separation and identification were challenging due to overlapping peaks in the spectra. In Figure 5f, the peaks for Cu could not be resolved. Similarly, in Figure 7f and Figure 8i the peaks for Cu between 0.67 and 1.5 MeV were not distinguishable. In Figure 7c, the group of peaks for In between 1 and 1.5 MeV were unresolved. In Figure 7i and Figure 8i, a main peak for Co was accompanied by relatively small peaks, which may have had minimal impact on its identification. In Figure 9b, the main peak for V was located in close proximity to low-intensity peaks, potentially not interfering with its identification.

The intensity of the counts of the KCl PGNA converter was not impaired by the addition of 20% of different materials at energies up to 1.2 MeV. While for energies above 1.6 MeV, the chlorine’s ability to detect neutrons increased, adding certain metals (Zn and Cd) may cause a general decrease in the peaks’ intensity, related directly to the decrease in the KCl amount to 80%. Other metals, V, Cu, and Co, increase the number of neutron interactions that led to their conversion to photons in the converter.

In general, Zn was found to be less effective for all tested neutron energies because no significant peaks were formed in the gamma spectrum.

## 5. Conclusions

The use of medical accelerators that produce fast neutrons as a byproduct rose in recent years, making the measurement of these neutrons increasingly vital. Neutrons can be measured using gamma radiation detectors, which are readily available. A neutron radiation measurement using the PGNA method was carried out using a gamma detector. Previous studies found that the use of Cl-35 as a PGNA converter is effective for fast neutron detection.

This study was conducted using MCNP Monte Carlo simulations in which different metals were added to KCl as a converter to improve the fast neutron detection efficiency. Up to 1.2 MeV, the KCl was less effective without the added metals, and from 1.6 MeV, certain metals caused a decrease in the efficiency of the KCl converter due to the high efficiency of chlorine.

We found that using a gamma radiation detector to measure fast neutrons had an appropriate detectability, and it is recommended that different metals be added to the potassium chloride salt according to varying neutron energies in order to obtain optimal efficiency for this type of detector. The neutron radiation measurement for several simulated energies using a gamma-ray detector was improved by adding different metals to the KCl converter.

Previous studies recommended the use of a converter composed of solutions. The physical process of preparing a converter comprised of solid KCl salt with other additional elements is relatively straightforward. The utilization of NaI(Tl) is a commonly adopted practice, and for the specified application, the total-count measurement mode is considered the most practical option.

## Figures and Tables

**Table 1:** Substances added to the KCl salt during the simulation and the cross-sections that were chosen for each substance.

Element	Cross Sections Series
Zinc (Zn)	.42C LLNL
Titanium (Ti)	.61C B-VI.8
Scandium (Sc)	.62C B-VI.8
Indium (In)	.60C B-VI.0
Copper (Cu)	.61C B-VI.8
Cadmium (Cd)	.42C LLNL
Vanadium (V)	.62C B-VI.8
Cobalt (Co)	.60C B-VI.0

**Table 2:** Gamma ray response of each material versus initial neutron energy.

Material	Neutron Energy (MeV)					
	0.02	1	1.2	1.6	2.2	3
**KCl + In**	Yes	No	Yes	Yes	No	No
**KCl + Cd**	Yes	No	No	No	No	No
**KCl + Ti**	Yes	No	Yes	Yes	No	No
**KCl + V**	No	Yes	Yes	Yes	Yes	Yes
**KCl + Cu**	No	Yes	Yes	Yes	Yes	Yes
**KCl + Co**	No	No	No	Yes	Yes	Yes
**KCl + Sc**	No	No	No	No	No	No
**KCl + Zn**	No	No	No	No	No	No

## Data Availability

The data presented in this study are available on request from the corresponding author.
